# Nasopharyngeal viral infection as a source of elevated fecal calprotectin: a diagnostic pitfall in intestinal inflammation

**DOI:** 10.1186/s13099-025-00780-7

**Published:** 2025-12-02

**Authors:** Chun-Ting Chen, Annemiek A. van der Eijk, Jolanda J.C. Voermans, Hanneke J. van Vuuren, Maikel P. Peppelenbosch, Vincent T. Janmaat

**Affiliations:** 1 Department of Gastroenterology and Hepatology, Erasmus MC—University Medical Center Rotterdam, PO Box 2040, Rotterdam, NL-3000 CA The Netherlands; 2 Department of Viroscience, Erasmus MC—University Medical Center Rotterdam, PO Box 2040, Rotterdam, NL-3000 CA The Netherlands

**Keywords:** Fecal calprotectin, Nasopharyngeal infection, Inflammatory bowel disease, Biomarker specificity, SARS-CoV-2, Diagnostic pitfall

## Abstract

Fecal calprotectin is widely used as a non-invasive biomarker for intestinal inflammation, particularly in inflammatory bowel disease (IBD), the most common clinical context for its use, where misinterpretation can lead to unnecessary invasive procedures or inappropriate therapy escalation. However, its specificity has been questioned due to elevated levels observed in non-intestinal conditions. Here, we investigated whether nasopharyngeal viral infections contribute to elevated fecal calprotectin, thereby confounding its diagnostic interpretation. Bioinformatic analysis revealed high constitutive expression of calprotectin subunits (S100A8/A9) in squamous epithelia of the upper aerodigestive tract compared with distal gut mucosa. To assess gastrointestinal stability of epithelial-derived calprotectin, healthy volunteers ingested porcine calprotectin, which was subsequently detected in feces, demonstrating resistance to intestinal degradation. In a retrospective cohort, patients with non-SARS-CoV-2 respiratory viral infections displayed significantly higher fecal calprotectin levels than SARS-CoV-2 patients (447 µg/g vs. 82 µg/g, *p* < 0.001), despite limited gastrointestinal involvement. Nasopharyngeal calprotectin levels did not differ significantly between viral etiologies, but in COVID-19 patients, fecal calprotectin positively correlated with nasopharyngeal calprotectin (*R* = 0.43, *p* = 0.008) and viral load (*R* = 0.13, *p* = 0.026). These findings suggest that calprotectin released from proximal epithelial tissues during viral infection can persist through the gastrointestinal tract and influence fecal calprotectin levels, underscoring the importance of contextual interpretation of this biomarker and the value of incorporating virological assessment into diagnostic workflows.

**Supplementary Information:**

The online version contains supplementary material available at 10.1186/s13099-025-00780-7.

## Introduction

Fecal calprotectin is widely regarded as a sensitive and specific biomarker for detecting intestinal inflammation, and it plays a pivotal role in the clinical management of IBD. It is primarily used to guide clinical decision-making in IBD, and elevated levels can trigger downstream consequences such as unnecessary treatment escalation or invasive investigations if misinterpreted. Because calprotectin levels correlate with neutrophilic infiltration of the intestinal mucosa, the test has become a mainstay in the non-invasive diagnosis and monitoring of IBD. Its use is now firmly embedded in both clinical trials [1]and routine medical practice [2], where it supports the assessment of disease activity, treatment response, and early prediction of relapse without immediate recourse to invasive endoscopic procedures. Elevated fecal calprotectin is typically interpreted as *bona fide* evidence of gastrointestinal inflammation. For instance, in patients suffering from diarrhea due to SARS-CoV-2 infection, elevated fecal calprotectin levels have been accepted as strong evidence that direct infection of the intestinal epithelium plays a central role in the pathogenesis of this condition [3]. However, non-intestinal sources of calprotectin, such as *Helicobacter pylori* infection, have also been reported to elevate fecal calprotectin, which acts as a potential confounder [4–6].

Biochemically, calprotectin is a heterodimeric complex of the S100 family of calcium-binding proteins—specifically S100A8 and S100A9—which are presumed to be released predominantly by activated neutrophils and macrophages during acute inflammation [7]. It also exists as a stable, biologically active heterotetrametric, which may contribute to its persistence and detectability in feces. Hence, its fecal concentration is thought to reflect the activity of these innate immune cells within the intestinal mucosa. Nonetheless, over the years, a partial disconnect has been observed between endoscopic inflammation and fecal calprotectin levels, raising the possibility that calprotectin might also originate from non-intestinal or non-phagocytic sources, a concept that has received relatively little attention in the literature [8].

**Fig. 1:**
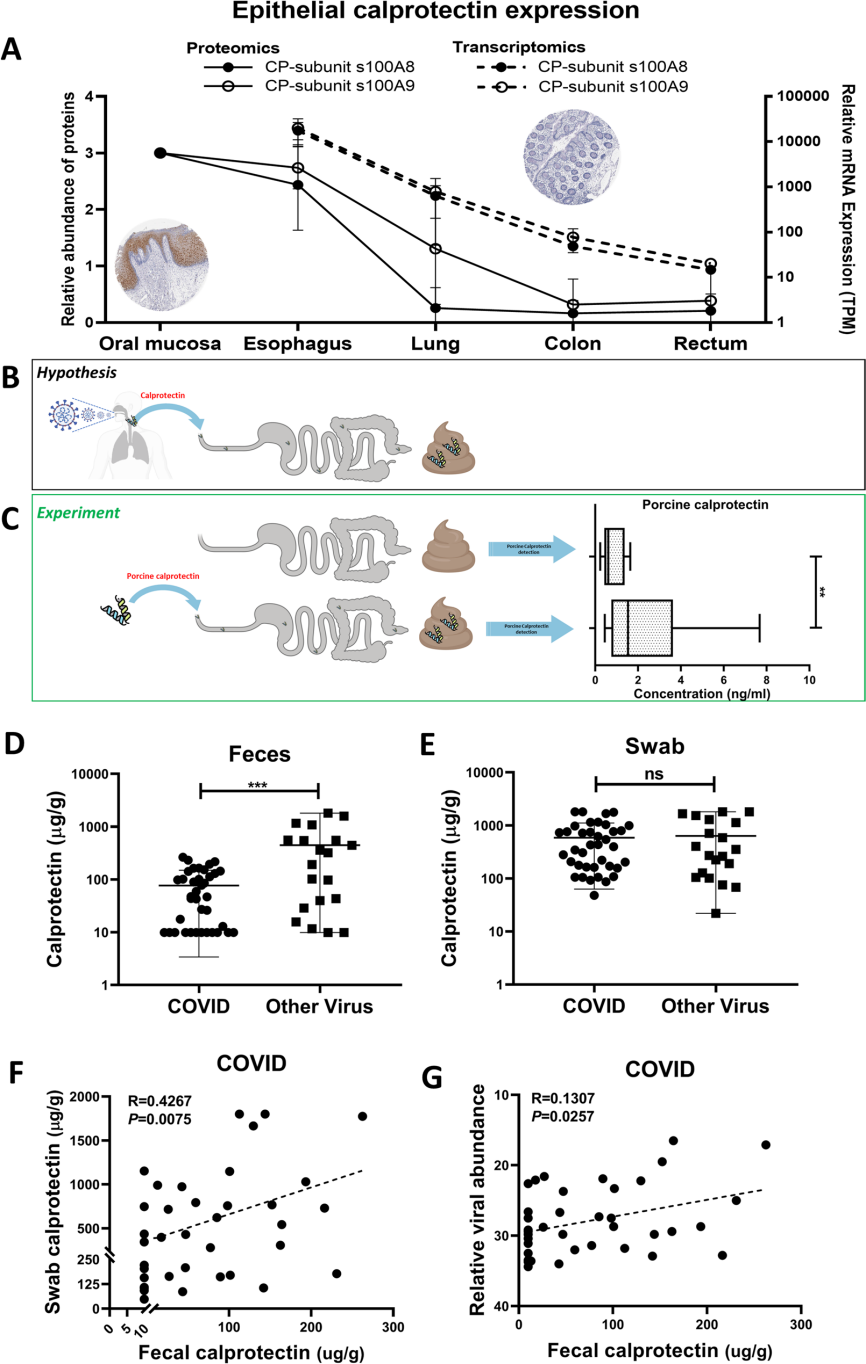
(**A**) Calprotectin expression in the upper respiratory tract and gastrointestinal tract. Expression of the two subunits of calprotectin (S100A/B) was assessed using proteomic, transcriptomic and immunohistochemistry (for methodology, see ref. 6). (**B**) Hypothesis as to the origin of fecal calprotectin in patients without intestinal inflammation. Viral infection of the upper respiratory and gastrointestinal tract provokes lysis of the squamous epithelium resulting release of calprotectin and its subsequent transport along the gut, finally resulting in elevated levels of fecal calprotectin. (**C**) A fecal sample was obtained from volunteers. Following 16 h of fasting, volunteers ingested 1cm3 of pig skin, which contains porcine calprotectin [14].For each volunteer, the latest two pre-ingestion and post-ingestion samples were compared using a species-specific ELISA. ** denotes *p* < 0.01 by Wilcoxon signed-rank test. (**D**) Fecal samples of patients with viral upper respiratory tract infection was probed for fecal calprotectin levels using Elisa. Results are separated with respect to viral etiology, COVID-19 and other (Adenovirus, Respiratory Syncytial virus or Human Para-Influenza Virus). ***denotes *p* < 0.001 by unpaired T test. (**E**) Protein was isolated from nasal swabs of the same patients as were investigated for nasopharyngeal fecal calprotectin levels using Elisa. (**F**) The results presented in D and E were investigated for correlation of nasal and fecal calprotectin levels for patients diagnosed with COVID-19. (**G**) For patients with COVID-19 viral titers were correlated to fecal calprotectin levels

Intriguingly, and in a different biological context, we have previously observed that upper gastrointestinal squamous epithelia, particularly in the esophagus, express high levels of calprotectin [9]. This expression appears to be constitutive and not necessarily inflammation-driven. Transcriptomic, proteomic, and immunohistochemical data was analyzed, specified in the material methods section under the *bioinformatics analysis of online database.* This analysis revealed that the subunits S100A8 and S100A9 are highly expressed in rostral epithelia, including the nasopharyngeal, oropharyngeal, and esophageal linings, whereas caudal gut segments such as the ileum and colon show much lower baseline expression levels (Fig. 1A). Based on these observations, we hypothesized that damage to proximal endodermal epithelium, for example due to upper respiratory tract infections, might release calprotectin from these tissues into the gastrointestinal tract. This calprotectin could then survive passage through the gut and be detected in feces—producing elevated fecal calprotectin levels that are not indicative of intestinal mucosal inflammation but still misinterpreted as such (see model in Fig. 1B).

## Materials and methods

### Sample preparation from COVID and other virus infection

#### Nasopharyngeal swab in universal transport medium (UTM)

Nasopharyngeal swab specimens were vortexed in UTM. The swab was removed from the UTM and measured using a pipette, after which the volume was adjusted to 4 ml with friction medium. Material extraction from the UTM tube was performed using a 5 ml syringe and blunt needle, followed by attachment of a 45 μm bacteriological filter. The filtered specimen was slowly dispensed into a 10 ml tube with a blue cap. Two aliquots were prepared containing 40% fetal bovine serum (FBS) and subsequently stored at -80 °C.

#### Feces

Fecal samples were transferred using a cotton swab into two 2 ml screw-cap tubes, filled up to the lower edge of the thread. No additives were used; pure material was stored.

### Calprotectin ELISA procedure

Calprotectin concentrations were analyzed according to the Erasmus MC Standard Operating Procedure Calprotectin [CALP] (Version 9, 2020) using the BÜHLMANN fCAL™ ELISA kit. A single stool sample per participant was collected and homogenized to minimize intra-sample variability. From the homogenized specimen, 0.1 g of feces was weighed into the base cap of a Smart-Prep extraction tube, and 4.9 mL of extraction buffer was added. The suspension was incubated for at least 10 min at room temperature, vortexed for one minute to ensure full homogenization, and centrifuged for 5 min at 3000 × g. Approximately 1 mL of the resulting supernatant was aliquoted and stored at − 20 °C until analysis.

Before measurement, samples were thawed and diluted 1:50 and 1:150 in incubation buffer. The ELISA plates, coated with calprotectin-specific monoclonal antibodies, were loaded with 100 µL of diluted samples, calibrators, and internal and external controls. After incubation and washing, horseradish peroxidase–conjugated detection antibody and TMB substrate were added sequentially. The enzymatic reaction was stopped with 0.25 M H₂SO₄, and absorbance was measured at 450 nm using a FLUOstar Omega microplate reader. Samples yielding values below 10 µg/g or above 1800 µg/g were reanalyzed for confirmation. Calibration and quality control were performed in each run according to the manufacturer’s instructions. The inter-run precision of the assay can vary by platform and conditions. According to the manufacturer, the inter-run coefficient of variation ranges from 5.3% to 12.8%. In our laboratory, the inter-run precision was determined to be 9.1% at an average fecal calprotectin concentration of 245 µg/g over 20 runs, indicating acceptable reproducibility for the study measurements.

### Porcine calprotectin experiment

Fecal samples were collected from four test subjects two to three times prior to the intervention. Each sample was weighed to ensure a mass 20 g, and was promptly stored at − 20 °C until further analysis. On the day of the intervention, porcine skin was prepared by weighing out 100 g. The porcine skin was pasteurized by poaching at 70 °C for 30 min. After pasteurization, the skin was seasoned liberally with a mixture of culinary spices. Each participant ingestied 1cm^3^ of pig skin for this experiment. Subsequent to porcine skin ingestion, sequential fecal samples were collected from each participant on four occasions, the latest two pre-ingestion and post-ingestion samples. These samples were also weighed (20 g per sample) and immediately stored at − 20 °C. After completion of sample collection, all specimens were assayed for levels of calprotectin and other target analytes utilizing a porcine skin-specific ELISA kit (CUSABIO, Wuhan, China), following the manufacturer’s instructions.

### Pig calprotectin (CALP) ELISA kit

Samples were rinsed with phosphate-buffered saline (PBS; 1×) and subsequently homogenized in 1 mL of PBS (1×). The homogenates were stored at − 20 °C overnight. To facilitate cell lysis, two freeze–thaw cycles were performed, after which the samples were centrifuged at 5000 × g for 5 min at 2–8 °C. The resulting supernatants were carefully collected into fresh microcentrifuge tubes and immediately subjected to analysis. Calprotectin concentrations were quantified using an enzyme-linked immunosorbent assay (ELISA) kit, following the manufacturer’s instructions. Absorbance values (optical density, O.D.) were measured with a microplate reader, and concentrations were calculated based on a standard calibration curve.

### Criteria for sample selection

Our study is a retrospective cohort study of patients admitted to the Erasmus MC. Cohorts consisting of the following patients were selected:

1. Swab positive for a SARS-CoV-2 upper respiratory tract virus (*n* = 38)
2. Swab positive for a non-SARS-CoV-2 upper respiratory tract virus (*n* = 20)

#### Inclusion criteria

Participants were eligible if they had a confirmed positive nasopharyngeal swab for SARS-CoV-2 or another viral pathogen during the study period. Fecal samples were eligible if collected within three days after the nasopharyngeal swab. Fecal sampling was performed at the discretion of the treating physicians to investigate gastrointestinal symptoms. Both swab and fecal samples were required to be collected and preserved according to established biobanking protocols, with subsequent testing performed within three days of sample collection.

#### Exclusion criteria

Participants were excluded if they had comorbidities known to affect gastrointestinal or immune function, including inflammatory bowel disease (IBD), active bacterial infections, celiac disease, gastrointestinal malignancies, or peptic ulcer disease.

### The bioinformatics analysis of online databases

Proteomic and transcriptomic datasets were obtained from The Human Protein Atlas (HPA) (https://www.proteinatlas.org/) FANTOM5 (https://fantom.gsc.riken.jp/5) and Genotype-Tissue Expression (GTEx) (https://gtexportal.org/home/) databases. Relative abundance in transcriptomic analyses refers to median transcripts per million (TPM) per tissue, while protein abundance values were obtained from GTEx-integrated proteomics resources, where normalized protein intensity values were available for selected tissues. HPA immunohistochemistry scores were used as qualitative validation of protein expression (none, low, medium, high).

### Statistical analysis

All statistical values were calculated using Statistical Product and Service Solutions (SPSS) 22.0 (Chicago, IL, USA). Experimental studies were analyzed by independent sample t-test to compare two groups. The Spearman rank correlation test was used to determine statistical correlations. Statistical significance was set at *P* < 0.05.

## Results

In order to assess the resistance of calprotectin to proteolytic degradation and chemical breakdown during transit through the gastric and intestinal environments, we conducted an ingestion study using porcine-derived calprotectin, which shares structural homology with human calprotectin but can be specifically detected using a pig-specific ELISA (Cusabio, CSB-Eq. 013485PI) [10, 7]. In a controlled experiment, healthy volunteers (*n* = 4) ingested a defined quantity of epithelial porcine calprotectin. Fecal samples collected before and after ingestion were assayed using the species-specific ELISA. The results demonstrated a significant and reproducible increase in fecal porcine calprotectin following ingestion (*p* < 0.01; Fig. 1C), providing direct experimental evidence that exogenous calprotectin from proximal epithelial sources can survive gastrointestinal transit and be recovered in the feces.

To further investigate the relevance of this mechanism under pathophysiological conditions, we performed a retrospective cohort study involving patients who had undergone nasopharyngeal swabbing and tested positive for either SARS-CoV-2 (*n* = 38) or non-SARS-CoV-2 respiratory viruses (*n* = 20). Fecal calprotectin levels were measured and compared between these groups. Interestingly, patients with non-SARS-CoV-2 respiratory viral infections exhibited markedly higher fecal calprotectin levels compared to COVID-19 patients (447 µg/g vs. 82 µg/g, respectively; *p* < 0.001; Fig. 1D), despite the absence of gastrointestinal symptoms in many cases.

Quantification of calprotectin in nasopharyngeal swabs from the same patients revealed no significant difference in calprotectin concentration between the SARS-CoV-2 and non-SARS-CoV-2 groups (Fig. 1E), suggesting that nasopharyngeal calprotectin levels per se are not virus-specific. Notably, no correlation between swab and fecal calprotectin was observed in the non-SARS-CoV-2 group, likely due to a smaller sample size, greater heterogeneity, and additional biological factors driving the elevated fecal calprotectin values in this group which were high in comparison to the values in the SARS-CoV-2 group (*Fig*. S1). Within the COVID-19 cohort, a positive correlation was observed between swab calprotectin levels and fecal calprotectin (*R* = 0.43; *p* = 0.008; Fig. 1F), indicating a potential dose-dependent relationship between epithelial calprotectin release and its gastrointestinal detection. Furthermore, fecal calprotectin levels in COVID-19 patients were found to correlate positively with viral load in the nasopharynx, as reflected by RT-PCR cycle threshold (Ct) values (*R* = 0.13; *p* = 0.0257; Fig. 1G). These findings support the view that nasopharyngeal viral activity contributes to systemic or luminal calprotectin levels and may represent a confounding factor in interpreting fecal calprotectin as a marker for gut inflammation.

## Discussion

Collectively, these results provide compelling evidence that upper respiratory tract viral infections can be a significant non-intestinal source of elevated fecal calprotectin. This has critical implications for clinical practice. Although the sensitivity of fecal calprotectin for detecting intestinal inflammation is well-established and approaches 100% in many studies [11], its specificity is known to be more variable [12]. Indeed, elevated calprotectin levels can also be seen in infectious enteritis, colorectal cancer, NSAID enteropathy, and—as shown here—extraintestinal infections. Despite widespread use of fecal calprotectin in clinical algorithms for IBD, the potential for false-positive results arising from non-intestinal sources has not been adequately addressed. Clinically, fecal calprotectin values above 250 µg/g are generally considered to indicate active intestinal inflammation, whereas results in the 100–250 µg/g “grey zone” often prompt repeat testing or further investigation [13]. Our data suggest that patients with upper respiratory viral infections may fall into this intermediate range or even exceed diagnostic thresholds, creating potential for false-positive interpretations. Such misinterpretation could lead to unnecessary endoscopic evaluation, escalation of therapy, or increased patient anxiety in the absence of true intestinal pathology. Our findings indicate that clinicians should exercise greater caution in interpreting elevated fecal calprotectin levels, particularly in patients who lack other clinical indicators of intestinal inflammation. Rather than recommending routine virological testing, a more appropriate approach may be to ask patients if symptoms of viral infections had occurred surrounding the moment of sampling. Subsequently, to re-test fecal calprotectin after resolution of upper respiratory symptoms, thereby reducing unnecessary invasive diagnostic or therapeutic interventions. The observed variability in non–SARS-CoV-2 viral cases likely reflects differences in viral tropism, epithelial involvement, and host inflammatory responses among pathogens, underscoring the broader limitation that the impact of upper respiratory tract infections on fecal calprotectin levels remains insufficiently understood. While we demonstrate a clear association, the extent to which different viral pathogens, infection severity, and host-related factors influence this phenomenon has not yet been established. Larger prospective studies will be needed to further delineate the scope and clinical implications of this effect. This work therefore expands our understanding of the biological origins of fecal calprotectin, demonstrating that calprotectin derived from proximal epithelial tissues—either ingested or released due to nasopharyngeal viral infection—can transmit the gastrointestinal tract and be detected in feces. Recognizing this phenomenon is essential for avoiding misdiagnosis and unnecessary procedures in patients with elevated fecal calprotectin but no other compelling clinical evidence of intestinal inflammation. Future diagnostic protocols may benefit from incorporating virological screening or serological markers to help distinguish genuine intestinal inflammation from systemic or proximal epithelial sources of calprotectin. As we refine the role of this biomarker in gastrointestinal medicine, contextual interpretation becomes key to ensuring its proper use in guiding clinical decisions.

## Supplementary Information

Supplementary Material 1.

## Data Availability

No datasets were generated or analysed during the current study.

## References

[1] Aharoni-Frutkoff Y et al. *Whole Food Diet Induces Remission in Children and Young Adults With Mild to Moderate Crohn’s Disease and Is More Tolerable Than Exclusive Enteral Nutrition: A Randomized Controlled Trial.* Gastroenterology, 2025. 10.1053/j.gastro.2025.06.011 40553742

[2] Hirten RP, et al. Physiological data collected from wearable devices identify and predict inflammatory bowel disease flares. Gastroenterology. 2025;168(5):939–e9515. 39826619 10.1053/j.gastro.2024.12.024 PMC12206379

[3] Cheung KS, et al. Gastrointestinal manifestations of SARS-CoV-2 infection and virus load in fecal samples from a Hong Kong cohort: systematic review and Meta-analysis. Gastroenterology. 2020;159(1):81–95. 32251668 10.1053/j.gastro.2020.03.065 PMC7194936

[4] Villalba-Davila P, et al. Helicobacter pylori infection is associated with significant elevations to fecal calprotectin, systemic inflammatory markers. J Pediatr Gastroenterol Nutr. 2025;80(4):617–22. 39831651 10.1002/jpn3.12464

[5] Aksoy OY, et al. Fecal calprotectin levels in Helicobacter pylori gastritis in children. Turk J Pediatr. 2020;62(6):986–93. 33372437 10.24953/turkjped.2020.06.010

[6] Rafeey M, Nikmanesh P, Javadzadeh F. Diagnostic value of fecal calprotectin in children with Gastritis, duodenitis and Helicobacter pylori. Int J Prev Med. 2022;13:107. 36247193 10.4103/ijpvm.IJPVM_507_20 PMC9564235

[7] Sejersen K, Eriksson MB, Larsson AO. Calprotectin as a biomarker for infectious diseases: A comparative review with conventional inflammatory markers. Int J Mol Sci, 2025. 26(13):6476. 10.3390/ijms26136476 PMC12249643 40650251

[8] Prentice RE, et al. Active inflammatory bowel disease on intestinal ultrasound during pregnancy is associated with an increased risk of adverse pregnancy and neonatal outcomes independent of clinical and biochemical disease activity. Gastroenterology. 2025;169(4):647–62. 40147617 10.1053/j.gastro.2025.03.016

[9] Janmaat VT, et al. HOXA13 in etiology and oncogenic potential of barrett’s esophagus. Nat Commun. 2021;12(1):3354. 34099670 10.1038/s41467-021-23641-8 PMC8184780

[10] Winogrodzki T, et al. TNF deltaare pigs: A translational crohn’s disease model. J Crohns Colitis. 2023;17(7):1128–38. 36821422 10.1093/ecco-jcc/jjad034 PMC10320488

[11] Holtman GA, et al. Diagnostic accuracy of fecal calprotectin for pediatric inflammatory bowel disease in primary care: A prospective cohort study. Ann Fam Med. 2016;14(5):437–45. 27621160 10.1370/afm.1949 PMC5394359

[12] Olivera PA, et al. Healthy First-Degree relatives from multiplex families vs simplex families have higher subclinical intestinal Inflammation, a distinct fecal microbial Signature, and harbor a higher risk of developing crohn’s disease. Gastroenterology. 2025;168(1):99–110. e2. 39236898 10.1053/j.gastro.2024.08.031

[13] Murray J, Kok KB, Ayling RM. Fecal Calprotectin Gastrointest Disease Clin Chem. 2023;69(7):699–710. 10.1093/clinchem/hvad051 37228058

[14] Abdi W, et al. An overview of S100 proteins and their functions in skin homeostasis, interface dermatitis conditions and other skin pathologies. Exp Dermatol. 2024;33(8):e15158. 39115029 10.1111/exd.15158

