# Supplementary material for: Nasopharyngeal viral infection as a source of elevated fecal calprotectin: a diagnostic pitfall in intestinal inflammation
Source: Gut Pathog. 2025 Dec 2;17:95. doi: 10.1186/s13099-025-00780-7 (PMC12673681; doi:10.1186/s13099-025-00780-7)
Supplement: Supplementary file 1 — Supplementary Material 1. [file 13099_2025_780_MOESM1_ESM.docx]

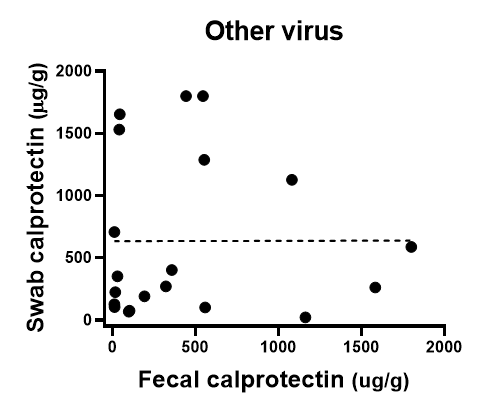

**Figure S1.**
Correlation analysis between nasopharyngeal and fecal calprotectin levels in patients infected with non–SARS-CoV-2 respiratory viruses (adenovirus, respiratory syncytial virus, or human parainfluenza virus)
